# The Benefit of Apparent Diffusion Coefficient in Evaluating the Invasiveness of Hepatocellular Carcinoma

**DOI:** 10.3389/fonc.2021.719480

**Published:** 2021-08-24

**Authors:** Mengyuan Jing, Yuntai Cao, Peng Zhang, Bin Zhang, Xiaoqiang Lin, Liangna Deng, Tao Han, Junlin Zhou

**Affiliations:** ^1^Department of Radiology, Lanzhou University Second Hospital, Lanzhou, China; ^2^Second Clinical School, Lanzhou University, Lanzhou, China; ^3^ Key Laboratory of Medical Imaging of Gansu Province, Lanzhou, China; ^4^Department of Radiology, Affiliated Hospital of Qinghai University, Xining, China; ^5^Department of Pathology, Lanzhou University Second Hospital, Lanzhou, China

**Keywords:** hepatocellular carcinoma, apparent diffusion coefficient, microvascular invasion, histological differentiation, Ki-67 antigen

## Abstract

**Background:**

This study aimed to evaluate hepatocellular carcinoma (HCC) invasiveness using the apparent diffusion coefficient (ADC).

**Methods:**

Eighty-one patients with HCC confirmed by pathology and examined by preoperative magnetic resonance imaging diffusion-weighted imaging from January 2015 to September 2020 were retrospectively analyzed. Clinical and pathological data were recorded. The minimum ADC (ADCmin), average ADC (ADCmean), and the ratio of ADCmean to normal-appearing hepatic parenchyma ADC (ADCnahp) were assessed. The associations between clinical information, ADC value, and HCC invasiveness (microvascular invasion [MVI], tumor differentiation, and Ki-67 expression) were evaluated statistically. Independent risk factors related to HCC invasiveness were screened using binary logistic regression, and the diagnostic efficiency was evaluated by the receiver operating characteristic curve and its area under the curve (AUC) value.

**Results:**

Tumor size was related to HCC MVI and tumor differentiation (*P* < 0.05). HCC MVI was associated with ADCmin, ADCmean, and the ADCmean-to-ADCnahp ratio (all *P* < 0.05) with AUC values of 0.860, 0.860, and 0.909, respectively. If these were combined with tumor size, the AUC value increased to 0.912. The degree of tumor differentiation was associated with ADCmin, ADCmean, and the ADCmean-to-ADCnahp ratio (all *P* < 0.05) with AUC values of 0.719, 0.708, and 0.797, respectively. If these were combined with tumor size, the AUC value increased to 0.868. Ki-67 expression was associated with ADCmin, ADCmean, and the ADCmean-to-ADCnahp ratio (all *P* < 0.05) with AUC values of 0.731, 0.747, and 0.746, respectively. Combined them, the AUC value increased to 0.763.

**Conclusions:**

The findings indicated that the ADC value has significant potential for the non-invasive preoperative evaluation of HCC invasiveness.

## Introduction

Liver cancer is the sixth most common cancer and the fourth leading cause of cancer death in the world, with a mortality rate greater than 8.2% (1). Due to the aging population and the imbalance of medical resources between urban and rural areas, the treatment of liver cancer has become a major burden in China (2). The number of annual liver cancer cases has increased from 258 000 in 1990 to 510 000 in 2017 (3).

The prognosis and treatment of patients with hepatocellular carcinoma (HCC) vary according to its invasiveness. For example, it has been reported that the presence of HCC microvascular invasion (MVI) is indicative of the likelihood of early recurrence after hepatectomy and liver transplantation (4, 5). For patients with MVI, tumor resection should be at a larger margin during operation or postoperative adjuvant chemoembolization can improve the long- term prognosis of the patients (6, 7). Ki-67 is an important cell proliferation marker. It has been demonstrated that different Ki-67 expression levels can predict the prognosis of patients with hepatitis B-related HCC and can also predict the efficacy of hepatectomy in patients with MVI and cancers in different Barcelona stages (8, 9). In addition, some studies have found that malignant tumors with a low degree of differentiation have a poor prognosis (10). Therefore, early identification of the invasiveness of HCC is beneficial for developing individualized treatment programs to improve patient prognosis.

The gold standard for determining the degree of HCC invasiveness is pathological and immunohistochemical investigation. However, accurate biological information cannot be obtained before diagnosis of HCC. Preoperative puncture biopsy has a high rate of misdiagnosis and delayed/missed diagnosis and may cause tumor implantation and intra-abdominal bleeding (11, 12). HCC MVI can only be found by histopathological examination of tumor specimens after the operation (13).

Diffusion-weighted magnetic resonance imaging (DWI) reflects the inherent differences in the dispersion movement of water molecules between tissues. The apparent diffusion coefficient (ADC) value is derived from DWI and has become a common imaging index used in clinical settings to quantify the biological behavior of tumors and to evaluate the curative effect (14, 15). Previous studies have shown that the ADC value can be used to predict early recurrence of liver cancer after radical resection (16) and to evaluate whether patients with liver cancer need additional treatment after the first transarterial chemoembolization procedure (17). However, there are few reports on the use of ADC values to evaluate the invasiveness of HCC (18). Therefore, this study aimed to evaluate the benefit of using the ADC as a non-invasive preoperative measure of HCC invasiveness.

## Materials and Methods

### Patients

**Figure 1:**
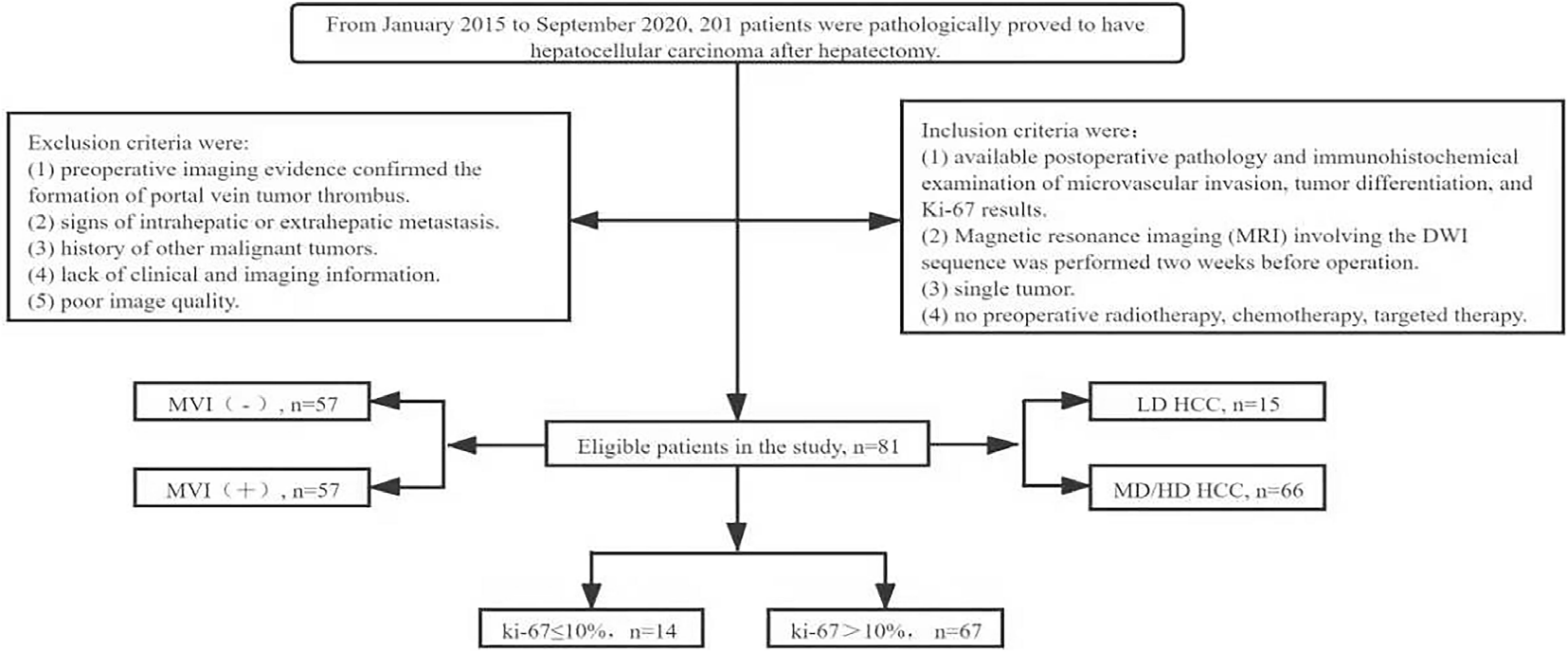
Flow chart of patient screening in the study. MVI, microvascular invasion; LD, low differentiation; MD, medium differentiation; HD, high differentiation; HCC, hepatocellular carcinoma.

This retrospective study was approved by the Ethics Committee of the Second Hospital of Lanzhou University (Ethical Board Approval No: 2020A-284), and the requirement for informed consent was waived. We collected clinical and imaging data for all patients who underwent hepatectomy and had a pathological diagnosis of HCC from January 2015 to September 2020. One hundred and twenty patients with histopathologically confirmed HCC were screened according to the following criteria. The inclusion criteria were (1) available postoperative pathology and immunohistochemical examination of MVI, tumor differentiation, and Ki-67 results; (2) magnetic resonance imaging (MRI) involving DWI sequences had been performed two weeks before the operation; (3) single tumor; and (4) no preoperative radiotherapy, chemotherapy, or other targeted therapy. Exclusion criteria were (1) preoperative imaging evidence confirming the formation of a portal vein tumor thrombus; (2) signs of intrahepatic or extrahepatic metastases; (3) history of other malignant tumors; (4) lack of clinical and imaging information; and (5) poor image quality. Finally, data were collected from a total of 81 patients who met the requirements (**Figure 1**). Clinical information such as age, sex, tumor size and location, history of hepatitis or liver cirrhosis, and the alpha-fetoprotein level was recorded.

According to the pathological and immunohistochemical results, MVI was divided into MVI (-) and MVI (+), the tumor differentiation was divided into moderately/well-differentiated and poorly differentiated, and Ki-67 was divided into low (Ki-67 ≤10%) and high (>10%) groups (19).

### Imaging Protocol

The Philips 3.0T superconducting MR (Ingenia 3.0T, Philips, Amsterdam, The Netherlands) and a 16-channel abdominal phased array coil were used. The scan ranged from the top of the diaphragm to the lower margin of the liver. The parameters of the MRI T1-weighted images were as follows: repetition time [TR]/echo time [TE], 3.7/1.32 ms; slice thickness, 5.0 mm; layer spacing, 1 mm; matrix, 220 × 193; field of view (FOV), 305 mm × 305 mm. For T2-weighted images (T2WI) the parameters were fat suppression (TR/TE of 716/75 ms), 6.5 mm slice thickness, 1 mm layer spacing, 132 × 117 matrix, 350 mm × 392 mm FOV. The parameters for DWI were TR/TE of 2443/75 ms, 6.5 mm slice thickness, 1 mm layer spacing, 132 × 117 matrix, 400 mm × 353 mm FOV, and number of excitation (NEX) of 2; diffusion gradients were applied in three orthogonal directions (b value = 0. 800 s/mm²). Axial-, sagittal-, and coronary-enhanced T1WI were obtained using gadopentetate dimeglumine (Gd-EOB-DTPA, Bayer Schering Pharma AG, Berlin, Germany) MRI contrast at 0.1 mmol/kg at a rate of 2.0 ml/s. 

### ADC Image Analysis

**Figure 2:**
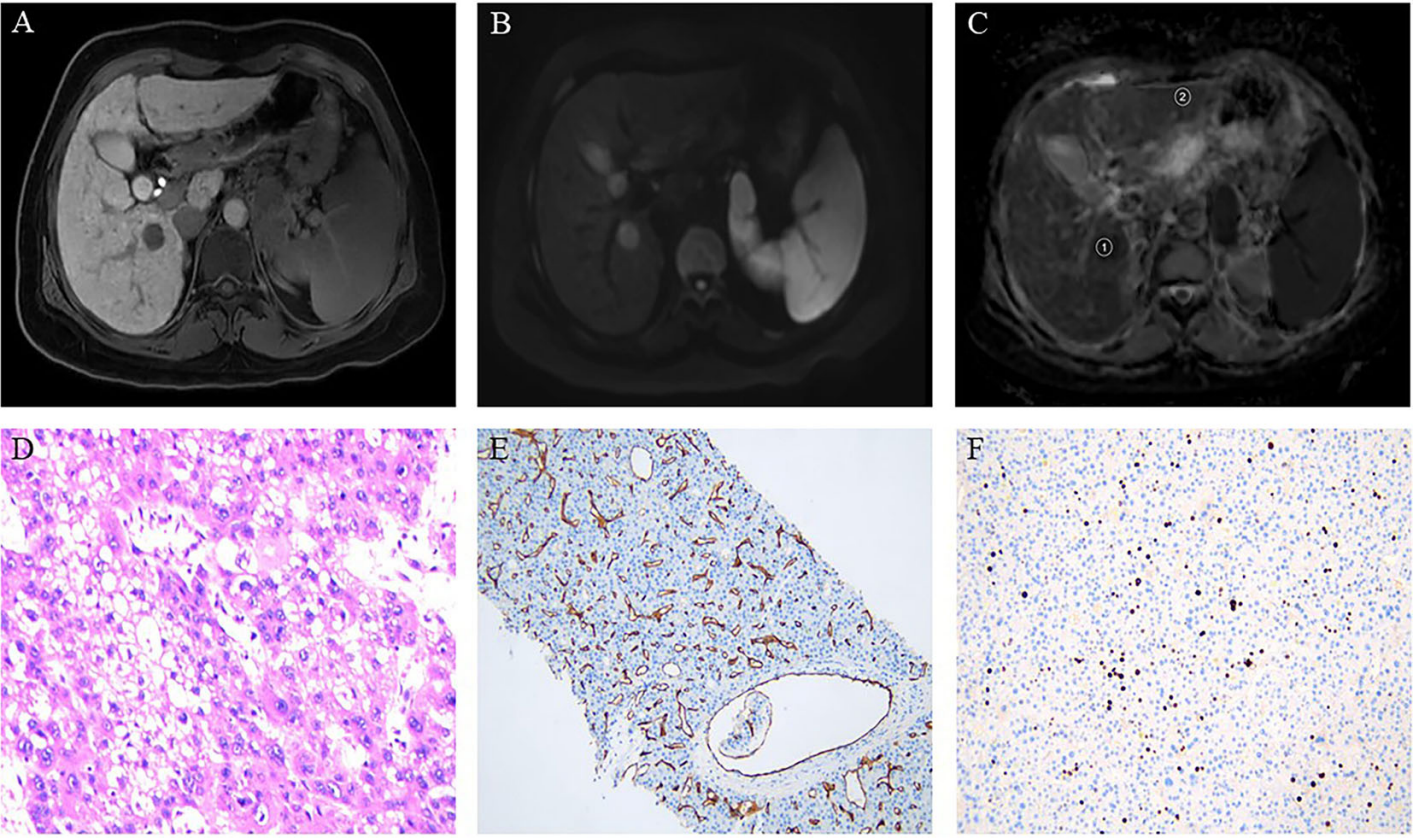
Hepatocellular carcinoma in a 49-year-old woman showing a round segment VI mass in the liver. **(A)** Axial lesions in the hepatobiliary phase showing low signal intensity relative to the hepatic parenchyma. **(B)** Diffusion-weighted imaging axial lesions showing high signal intensity. **(C)** Apparent diffusion coefficient (ADC) map showing the regions of interest used to determine the average ADC (1), and the ADC in normal-appearing hepatic parenchyma (2). **(D)** Pathology revealing poorly differentiated hepatocellular carcinoma (HE×400). **(E)** Immunohistochemistry showing microvascular invasion. **(F)** Immunohistochemistry showing high proliferative activity of tumor cells with approximately 20% Ki-67 expression.

**Figure 3:**
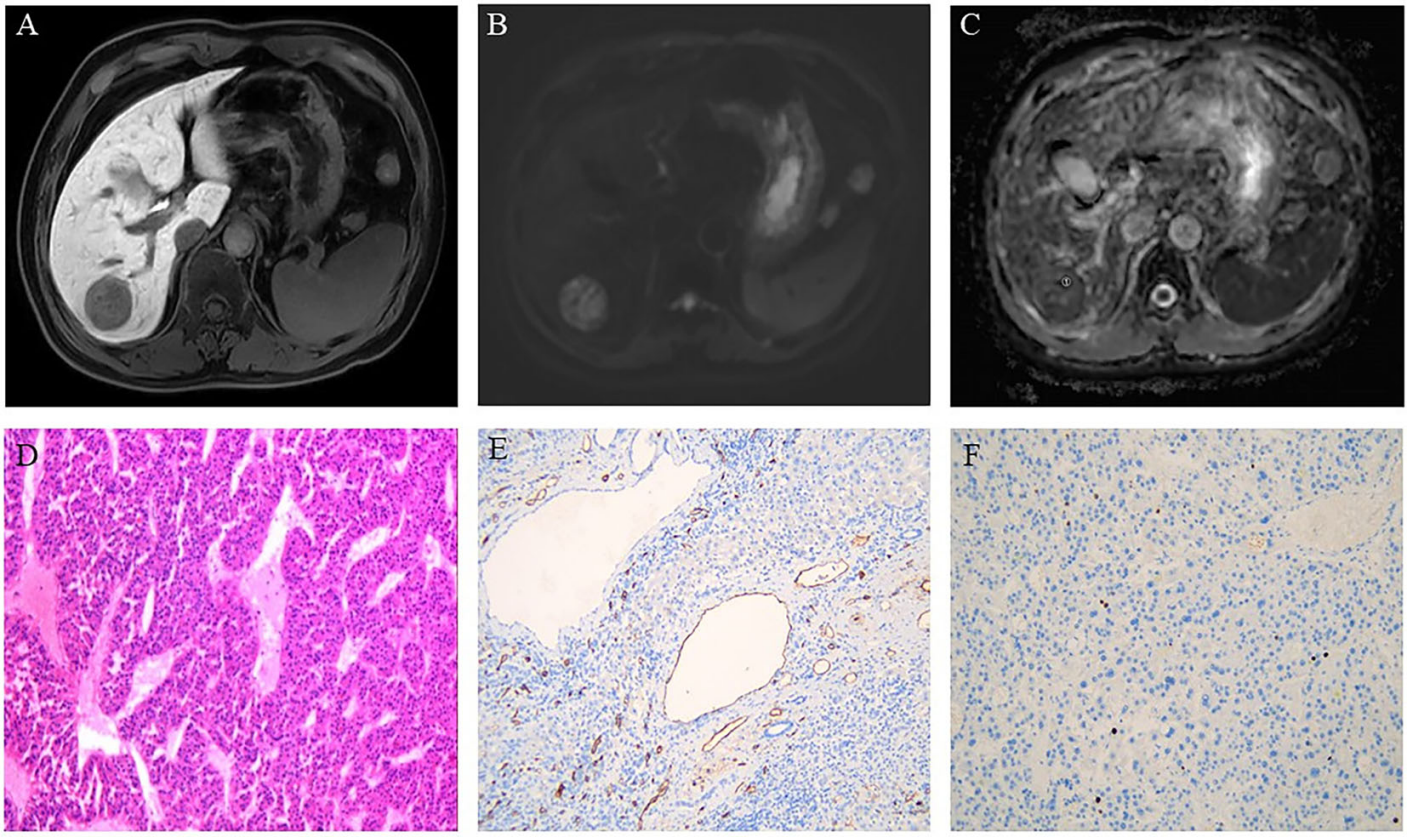
Hepatocellular carcinoma in a 61-year-old man showing a round segment VI mass in the liver. **(A)** Axial lesions in the hepatobiliary phase showing low signal intensity relative to the hepatic parenchyma. **(B)** Diffusion-weighted images of axial lesions showing relatively high signal intensity. **(C)** Apparent diffusion coefficient (ADC) map showing the region of interest used to determine the minimum ADC (1). **(D)** Pathology revealing moderately differentiated hepatocellular carcinoma (HE×100). **(E)** Immunohistochemistry showing an absence of microvessel invasion by the tumor tissue. **(F)** Immunohistochemistry showing low proliferative activity of the tumor cells with approximately 3% Ki-67 expression.

In ADC maps, the ADC measurements were analyzed blindly by three radiologists with 25, 10, and 6 years of experience in abdominal MRI diagnosis, respectively. The regions of interest (ROIs) were drawn in the solid portions of the tumor, avoiding the blood vessels, cystic degeneration, necrosis, calcification, and bleeding areas of the tumor as indicated by MRI-enhanced images and DWI images. The ROIs of the minimum ADC (ADCmin) were placed into the visually perceived lowest ADC parts of the solid tumor where the diffusion of the lesion was most limited in the DWI images (20). Subsequently, according to the size of the lesion, one large ROI [the average ADC (ADCmean)] was placed in the solid portion of the tumor to cover the largest axial solid tumor cross-section. Finally, at the same level as ADCmean ROIs, ROIs with the same area as the ADCmean ROIs were used as comparative ADCs and placed in the normal-appearing hepatic parenchyma (ADCnahp) farthest from the lesion while avoiding the hepatic vessels. The ADCmean divided by the comparative ADC value was defined as the ADCmean-to-ADCnahp ratio. The average values of the measurements of three radiologists were used as the final results for follow-up analysis. **Figures 2**, **3** show the MRI images and pathological immunohistochemical images of patients with HCC while showing the ROIs used to determine the ADCmean, ADCmean-to-ADCnahp ratio, and ADCmin.

### Statistical Analysis

SPSS Statistics 23.0 (IBM Corp. Armonk, NY, USA) was used for all data analysis. *P* < 0.05 (bilateral) was used to indicate statistical significance. The chi-square or Fisher’s exact tests were used to analyze the correlations between sex, tumor size and location, history of hepatitis or liver cirrhosis, and alpha-fetoprotein and HCC MVI, tumor differentiation, and Ki-67. Student’s t-test or the Mann-Whitney U test were used to analyze the correlations between age, ADCmin, ADCmean, and ADCmean-to-ADCnahp ratio, and HCC MVI, tumor differentiation, and Ki-67. All continuous variables were expressed as mean ± standard deviation (SD). Categorical variables were represented as N (%). Univariate and multivariate analyses were used to screen the clinical features and imaging parameters that were highly associated with pathological features. Our study used the area under the curve (AUC) values to evaluate the diagnostic efficacy of ADCmin, ADCmean, and the ADCmean-to-ADCnahp ratio in evaluating HCC MVI, tumor differentiation, and Ki-67

## Results

### Clinical and Pathological Features of HCC

As shown in **Table 1**, this study included 81 patients with HCC, including 62 males and 19 females [mean age 52 (29–75) years)]. Among them, there were 57 patients without MVI and 24 patients with MVI. A total of 15 patients had poorly differentiated HCC and 66 patients had moderately/well-differentiated HCC. A total of 14 patients had tumors with Ki-67 ≤10% and 67 had tumors with Ki-67 >10%. There were no significant differences in other clinical factors in the above groups (P>0.05, **Table 1**). Tumor size was related to MVI and HCC tumor differentiation (P<0.05) but not to Ki-67 expression (P>0.05).

**Table 1:** Clinical and pathological features of hepatocellular carcinoma.

Variable	MVI (-)	MVI (+)		PD	MD/WD		ki-67 ≤ 10%	ki-67>10%	
	n = 57	n = 24	*P*-Value	n = 15	n = 66	*P*-Value	n = 14	n = 67	*P*-Value
Age (years)	52.46 ± 9.48	51.67 ± 7.45	0.718	51.93 ± 7.50	52.29 ± 9.23	0.890	55.50 ± 9.51	51.54 ± 8.67	0.130
Gender			0.832			0.089			0.844
Male	44 (77.2)	18 (75.0)		14 (93.3)	48 (72.7)		11 (78.6)	51 (76.1)	
Female	13 (22.8)	6 (25.0)		1 (6.7)	18 (27.3)		3 (21.4)	16 (23.9)	
Size (cm)			0.002			0.001			0.152
≤3	33 (57.9)	5 (20.8)		1 (6.7)	37 (56.1)		9 (64.3)	29 (43.3)	
>3	24 (42.1)	19 (79.2)		14 (93.3)	29 (43.9)		5 (35.7)	38 (56.7)	
Tumour location			0.971			0.183			0.941
Left	24 (42.1)	10 (41.7)		4 (26.7)	30 (45.5)		6 (42.9)	28 (41.8)	
Right	33 (57.9)	14 (58.3)		11 (73.3)	36 (54.5)		8 (57.1)	39 (58.2)	
Hepatitis			0.126			0.368			0.069
No	13 (22.8)	2 (8.3)		4 (26.7)	11 (16.7)		5 (35.7)	10 (14.9)	
Yes	44 (77.2)	22 (91.7)		11 (73.3)	55 (83.3)		9 (64.3)	57 (85.1)	
Cirrhosis			0.636			0.109			0.924
No	16 (28.1)	8 (33.3)		7 (46.7)	17 (25.8)		4 (28.6)	20 (29.9)	
Yes	41 (71.9)	16 (66.7)		8 (53.3)	49 (74.2)		10 (71.4)	47 (70.1)	
AFP			0.083			0.260			0.138
<400	26 (45.6)	6 (25.0)		4 (26.7)	28 (42.4)		8 (57.1)	24 (35.8)	
≥400	31 (54.4)	18 (75.0)		11 (73.3)	38 (57.6)		6 (42.9)	43 (64.2)	

MVI, microvascular invasion; PD, poorly differentiated; MD, moderately differentiated; WD, well differentiated; AFP, alpha-fetoprotein.

### ADC Value and Pathological Features of HCC

**Table 2** shows the ADCmin, ADCmean, and ADCmean-to-ADCnahp ratios in relation to HCC invasiveness. The HCC with MVI group had lower ADCmin, ADCmean, and ADCmean-to-ADCnahp ratios (all *P* < 0.05) than the HCC without MVI group. Poorly differentiated HCC also had lower ADCmin, ADCmean, and ADCmean-to-ADCnahp ratios (all *P*<0.05) than the moderately/well-differentiated group. The HCC group with low Ki-67 expression had higher ADCmin, and ADCmean-to-ADCnahp ratios (all *P*<0.05) than the group with high Ki-67 expression.

**Table 2:** ADC value and pathological features of hepatocellular carcinoma.

	MVI(-)	MVI(+)	*P*-Value	PD	MD/WD	*P*-Value	Ki-67 ≤ 10%	ki-67 > 10%	*P*-Value
ADCmin (10^-3^mm^2^/s)	1.12 ± 0.20	0.87 ± 0.15	0.000	0.92 ± 0.18	1.08 ± 0.22	0.009	1.19 ± 0.24	1.02 ± 0.20	0.021
ADCmean (10^-3^mm^2^/s)	1.16 ± 0.20	0.90 ± 0.16	0.000	0.96 ± 0.18	1.11 ± 0.22	0.015	1.24 ± 0.24	1.05 ± 0.20	0.003
ADCmean-to-ADCnahp ratio (10^-3^mm^2^/s)	1.10 ± 0.19	0.81 ± 0.13	0.000	0.84 ± 0.14	1.06 ± 0.22	0.000	1.16 ± 0.21	0.99 ± 0.21	0.005

MVI, microvascular invasion. PD, poorly differentiated; MD, moderately differentiated; WD, well differentiated; ADCmin, minimum ADC; ADCmean, average ADC; ADCmean-to-ADCnahp ratio, ratio of average ADC to normal-appearing parenchyma ADC.

### Receiver Operating Characteristic Curve and Area Under the Curve for Judging HCC Invasiveness

**Figure 4:**
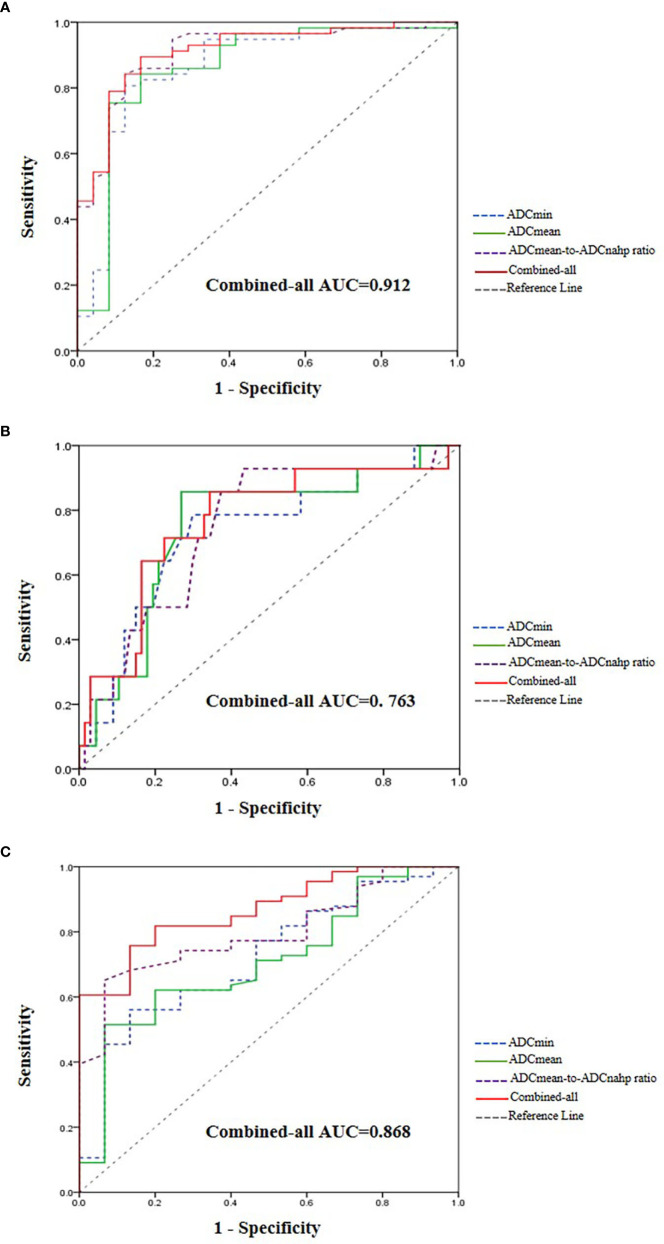
Receiver operating characteristic curve and area under the curve for judging hepatocellular carcinoma (HCC) microvascular invasion **(A)**, tumor differentiation **(B)**, and Ki-67 expression **(C)**.

For the prediction of HCC with MVI, the AUC values of ADCmin, ADCmean, and the ADCmean-to-ADCnahp ratio were 0.860, 0.860, and 0.909, respectively (**Table 3**). According to the ROC curve analysis, the best critical points of ADCmin, ADCmean, and the ADCmean-to-ADCnahp ratio were 0.97×10^-3^ mm^2^/s, 0.97×10^-3^ mm^2^/s, and 0.94×10^-3^mm^2^/s, respectively. Binary logistic regression combined with ADCmin, ADCmean, the ADCmean-to-ADCnahp ratio, and tumor size was the best method for predicting HCC microvascular invasion (AUC, 0.912; sensitivity, 83.3%; specificity, 89.5%; **Figure 4A**).

**Table 3:** ADC value, predictive probability and pathological features of hepatocellular carcinoma.

		AUC	95%CI	Cutoff value	Sensitivity	Specificity
MVI	ADCmin	0.860	0.760 - 0.960	0.97 (10^-3^mm^2^/s)	80.7%	87.5%
	ADCmean	0.860	0.756 - 0.963	0.97 (10^-3^mm^2^/s)	84.2%	83.3%
	ADCmean-to-ADCnahp ratio	0.909	0.756 - 0.963	0.94 (10^-3^mm^2^/s)	84.2%	87.5%
	Combined-all	0.912	0.844 - 0.979	0.45	83.3%	89.5%
Tumor differentiation	ADCmin	0.719	0.582 - 0.856	1.06 (10^-3^mm^2^/s)	56.1%	86.7%
	ADCmean	0.708	0.570 - 0.845	1.14 (10^-3^mm^2^/s)	51.5%	93.3%
	ADCmean-to-ADCnahp ratio	0.797	0.693 - 0.902	0.99 (10^-3^mm^2^/s)	65.2%	93.3%
	Combined-all	0.868	0.784 - 0.952	0.83	75.8%	86.7%
Ki-67	ADCmin	0.731	0.581 - 0.880	1.13 (10^-3^mm^2^/s)	78.6%	70.1%
	ADCmean	0.747	0.603 - 0.892	1.17 (10^-3^mm^2^/s)	85.7%	73.1%
	ADCmean-to-ADCnahp ratio	0.746	0.608 - 0.884	1.03 (10^-3^mm^2^/s)	92.9%	56.7%
	Combined-all	0.763	0.619 - 0.908	0.83	65.7%	85.7%

Combined-all: 1) Binary logistic regression analysis was used to analyze ADC parameters [minimum ADC (ADCmin), average ADC (ADCmean), and ratio of average ADC to normal-appearing parenchyma ADC (ADCmean-to-ADCnahp ratio)] and tumor size in relation to microvascular invasion (MVI) and tumor differentiation. 2) Binary logistic regression analysis was used to combine ADC parameters related to Ki-67 (ADCmin, ADCmean, ADCmean-to-ADCnahp ratio).

For the prediction of HCC tumor differentiation, the AUC values of ADCmin, ADCmean, and the ADCmean-to-ADCnahp ratio were 0.719, 0.708, and 0.797, respectively (**Table 3**). According to the ROC curve analysis, the best critical points of ADCmin, ADCmean, and the ADCmean-to-ADCnahp ratio were 1.06×10^-3^ mm^2^/s, 1.14×10^-3^ mm^2^/s, and 0.99×10^-3^ mm^2^/s, respectively. Binary logistic regression combined with ADCmin, ADCmean, the ADCmean-to-ADCnahp ratio, and tumor size performed the best for predicting the degree of HCC differentiation (AUC, 0.868; sensitivity,75.8%; specificity, 86.7%; **Figure 4B**).

For the prediction of Ki-67 expression in HCC, the AUC values of ADCmin, ADCmean, and the ADCmean-to-ADCnahp ratio were 0.731, 0.747, and 0.746, respectively (**Table 3**). According to the ROC curve analysis, the best critical points of ADCmin, ADCmean, and the ADCmean-to-ADCnahp ratio were 1.13×10^-3^ mm^2^/s, 1.17×10^-3^ mm^2^/s, and 1.03×10^-3^ mm^2^/s, respectively. Binary logistic regression combined with ADCmin, ADCmean, and the ADCmean-to-ADCnahp ratio was the best for predicting HCC Ki-67 expression (AUC, 0.763; sensitivity, 65.7%; specificity, 85.7%; **Figure 4C**).

## Discussion

In this study, we evaluated the correlation between the ADC value and HCC invasiveness. The results showed that ADCmin, ADCmean, and the ADCmean-to-ADCnahp ratio were significantly related to HCC invasiveness. When these three parameters combined with clinical features associated with HCC invasiveness, the accuracy of the biological evaluation of HCC was improved. Our study showed that tumor size was associated with both MVI and tumor differentiation, with larger tumors having a higher probability of MVI but a lower degree of differentiation; however, tumor size had no effect on Ki-67 expression. These results are consistent with previously reported findings (19, 21–23). Pawlik et al. (23) found that 25% of tumors 3 cm or smaller were associated with MVI, compared with 40% of lesions 3.1 to 5 cm, 55% of lesions 5.1 to 6.5 cm, and 63% of lesions 6.5 cm or larger. In addition, 36% of tumors 5 cm or smaller were high-grade, compared with 54% of tumors 5.1 to 6.5 cm. However, Li et al. (19) did not find a significant correlation between tumor size and the degree of differentiation, probably because all the participants in their study had tumors below 3 cm in size.

DWI is an MRI technique based on changes in water diffusion and can thus provide information about tissue microstructure. The ADC value derived from DWI can improve the quantification of tumor biology (24). Our study found that there was a correlation between the ADC value and the invasiveness of HCC, which is consistent with previous reports (19, 25, 26). The ADCmin and ADCmean of HCC with non-MVI, showing moderate to good differentiation, and low Ki-67 expression were higher than those of HCC tumors associated with MVI, poor differentiation, and high Ki-67 expression, which is consistent with previous reports (19, 25, 26). Previous studies have shown that tumor cell density and the nuclear/cytoplasmic (N/C) ratio of HCC with MVI, lower degree of differentiation, and higher Ki-67 expression may lead to a more obvious diffusion limitation, resulting in the lower ADC values (27–32). Regarding the relationship between the differentiation of HCC and the ADCmean, there were some differences between this study and other studies due to differences in grouping (29–32). However, previous studies have shown that a lower ADC value indicated a lower degree of differentiation (27–32). This is because HCC differentiation is mainly dependent on mitotic activity and the N/C ratio. As the mitotic activity and N/C ratio increase in association with the reduction in differentiation, the rate of water diffusion decreases, leading to a decrease in the ADCmean (30). To verify the repeatability and reliability of the research results, we further compared the effectiveness of the ADCmin, ADCmean, and ADCmean-to-ADCnahp ratio in evaluating HCC invasiveness by expanding the sample size. We found that the ADCmean-to-ADCnahp ratio was better able to distinguish MVI and HCC tumor differentiation than the ADCmin and ADCmean. The ability of the ADCmean-to-ADCnahp ratio to evaluate Ki-67 expression was similar to that of the ADCmean, but higher than that of the ADCmin. To our knowledge, there have been no previous studies on the use of the ADCmean-to-ADCnahp ratio to evaluate the invasiveness of HCC although it has been found to be of great value in assessing brain tumors (20). The ADCmean-to-ADCnahp ratios can accurately reflect the differences in diffusion between lesions and normal tissues and are not easily affected by differences in scanning devices or scanning settings, so they may be more suitable to evaluate abnormal diffusion (33).

The disagreement between the three radiologists may have affected the results of the measurements. However, using the intraclass correlation coefficient (ICC), good agreements were obtained between the three radiologists for the measurements of ADCmin (ICC=0.823, P<0.001), ADCmean (ICC=0.831, P<0.001), and the ADCmean-to-ADCnahp ratio (ICC=0.805, P<0.001). The evaluation accuracy of HCC invasiveness was significantly improved by combining the ADCmin, ADCmean, ADCmean-to-ADCnahp ratio, and tumor size; the AUC values were 0.912, 0.868, and 0.763, respectively. The results showed that the ADC value combined with tumor size can be used as a non-invasive method to evaluate the invasiveness of HCC before surgery, providing valuable information for the personalized treatment of HCC and thus improving patient prognosis.

This study has some limitations. Firstly, only three common levels of HCC invasiveness were used, and the relationship between the ADC value and additional levels of invasiveness should be further explored in the future. Secondly, this study was a single-center study, and multicenter studies with larger sample sizes are needed to further verify the research results. Thirdly, the ADC value will vary in response to different machines and scanning parameters, the DWI of the multi-b-value intravoxel incoherent motion may reduce the variability as much as possible, and further studies are required. Finally, although the radiologists were blinded, some cases may have been previously discussed by the very same radiologist analyzing the ADC value, which might have biased the results. However, the probability of this happening was extremely low, and we used an average of the measurements of three different radiologists to reduce the impact of this possible situation on the results.

## Conclusions

In conclusion, the ADC value is of great clinical benefit in the non-invasive preoperative evaluation of HCC invasiveness and has great potential as a clinical tool for personalized clinical treatment.

## Data Availability Statement

The raw data supporting the conclusions of this article will be made available by the authors, without undue reservation.

## Author Contributions

MJ, YC, PZ, BZ, XL, LD and TH: drafting and refining the manuscript. MJ, YC, and JZ: critical reading of the manuscript. All authors contributed to the article and approved the submitted version.

## Funding

This research was funded by the National Natural Science Foundation of China, grant number 81772006, 82071872.

## Conflict of Interest

The authors declare that the research was conducted in the absence of any commercial or financial relationships that could be construed as a potential conflict of interest.

## Publisher’s Note

All claims expressed in this article are solely those of the authors and do not necessarily represent those of their affiliated organizations, or those of the publisher, the editors and the reviewers. Any product that may be evaluated in this article, or claim that may be made by its manufacturer, is not guaranteed or endorsed by the publisher.
